# Prophylactic Tranexamic Acid Use in Orthognathic Surgery: A Systematic Review and Meta-analysis

**DOI:** 10.1007/s00266-025-04738-7

**Published:** 2025-03-10

**Authors:** Hooman Baghaie, Khilan Shukla, Jennifer Stone, Omar Breik, Zachary Munn

**Affiliations:** 1 JBI, School of Public Health, The University of Adelaide, Adelaide, SA Australia; 2 School of Medicine, Griffith University, Brisbane, QLD Australia; 3 Department of Oral and Maxillofacial Surgery, Royal Brisbane and Women’s Hospital, Brisbane, QLD Australia; 4 Adelaide GRADE Centre, School of Public Health, University of Adelaide, Australia, Adelaide, Australia; 5 School of Dentistry, University of Queensland, Brisbane, ALD Australia; 6 Health Evidence Synthesis, Recommendations and Impact (HESRI), School of Public Health, University of Adelaide, Adelaide, Australia

**Keywords:** Orthognathic surgery, Maxilla, Mandible, Bleeding, Tranexamic acid

## Abstract

**Background:**

The objective of this systematic review and meta-analysis was to assess the effects of tranexamic acid (TXA) on bleeding and thromboembolic events in orthognathic surgery out.

**Methods:**

Three electronic databases (PubMed, Web of Science, and Cochrane Library) were searched until 01/06/2024.

**Results:**

Nine randomised controlled trials and two cohort studies were included for pooled analysis. Compared with the control group, the TXA group showed a mean reduction in intraoperative blood loss of 171.30 ml (p < 0.00001; 95% CI, 197.58-145.02 ml). Systemic tranexamic acid use was associated with reduced operative time by a mean of14.5 minutes (p < 0.0001; 95% CI, 20.89-8.02 mins) and reduction in the need for transfusion OR= 0.33 (p < 0.01; 95% CI, 0.14-0.77).

**Conclusion:**

This systematic review and meta-analysis supports the prophylactic use of tranexamic acid during orthognathic surgery in reducing blood loss, operative time and the risk of needing blood transfusions without increasing risk of thromboembolic complications.

**Level of Evidence I:**

This journal requires that authors assign a level of evidence to each article. For a full description of these Evidence-Based Medicine ratings, please refer to Table of Contents or the online Instructions to Authors  www.springer.com/00266.

## Introduction

Orthognathic surgery (OS) is a common elective surgery for correcting dentomaxillofacial deformities. It entails performing mandibular and/or maxillary osteotomies and fixation of bony segments into ideal functional and cosmetic relationships [1]. These elective procedures typically result in significant bleeding, with historic blood loss of up to 5000ml [2]. The mean blood loss in single or bimaxillary orthognathic surgery is 800ml, with reports of up to 40% of patients receiving postoperative blood transfusions [3]. Various tools and techniques have been trialled in minimising bleeding which can be categorised into: surgical techniques [4–6], physiological modifications [7–12], or pharmacological adjuncts [13]. The most widely studied pharmacological adjunct in reducing blood loss in surgery is tranexamic acid (TXA).

Tranexamic acid is a synthetic amino acid that reversibly inhibits plasminogen activation, which in turn prevents the breakdown of microthrombi and reduces bleeding. The clinical effect of TXA is well documented with a 30% reduction in blood loss across various surgeries, including cardiothoracic, orthopaedic, obstetrics and gynaecology, as well as in general and maxillofacial trauma [14–17]. Though widely used and validated to limit blood loss in other surgical procedures; its use is not yet considered gold standard in orthognathic surgery despite several randomised control trials [18–20]. The aim of this systematic review and meta-analysis was to explore: *What is the effectiveness of prophylactic tranexamic acid versus saline or no intervention on blood loss in healthy patients receiving orthognathic surgery?*

## Materials and Methods

### Protocol and Registration

The systematic review was conducted in accordance with the JBI methodology for systematic reviews of effectiveness evidence [21]. The review protocol was registered in PROSPERO international prospective register of systematic reviews (CRD42022314403). The Preferred Reporting Items for Systematic Reviews and Meta-analyses (PRISMA 2020) [22] was used to guide the reporting of this review. The protocol was published in a peer-reviewed journal prior to conducting the review [23]. The detailed methods are available for reproduction [24].

### Eligibility Criteria

#### Inclusion

1. Population: Healthy patients undergoing orthognathic surgery (defined as minimum of Le Fort I maxillary osteotomy and/or variations of bilateral mandibular ramus osteotomies).
2. Intervention: Prophylactic administration of perioperative (topical or systemic) TXA.
3. Comparator: Saline or no treatment.
4. Outcome: Bleeding as measured by intraoperative blood loss, change in haemoglobin and/or haematocrit and need for blood transfusion. Secondary outcomes included operative time, duration of stay and adverse effects.
5. Study Design: Randomised controlled trials (RCTs) and pseudo-randomised controlled trials, published and unpublished, without language restrictions.

#### Exclusion

1. Patients with pre-existing coagulopathies or taking anticoagulant/antiplatelet medications.
2. Studies which included concurrent or additional surgical procedures to orthognathic surgery (e.g. rhinoplasty)
3. Non-RCTs, cohort studies, case reports, case series, reviews, abstracts, systematic reviews, expert opinions and animal studies

### Information Sources, Search Strategy and Study Selection

A librarian was used to develop the search strategy. The databases searched included PubMed, Embase and Cochrane library (including CENTRAL) [25]. Each database was screened from the date of its inception. The search strategy included published and unpublished studies, without language restrictions. An initial limited search of PubMed was undertaken followed by an analysis of the text words contained in the title and abstract, and index terms used to describe the relevant articles. A second search using all identified keywords and index terms was then undertaken across the three included databases. The reference lists of all included reports that underwent risk of bias assessment were searched for additional studies that may have been missed. Finally, the World Health Organisation International Clinical Trial Registry Platform and Clinicaltrials.gov database were searched for unpublished trials. The authors of any proposed or protocolled projects were contacted for further information if required.

### Data Extraction and Synthesis

Data were extracted from included studies by two independent reviewers using Covidence (Veritas Health Innovation, Melbourne, Australia). Data extracted included study details, participant characteristics, intervention and control details, outcome details and data. The information extracted was compared between the two reviewers and any disagreements were resolved through discussion.

### Risk of Bias and Assessment of Intervention Effect

Eligible studies were independently critically appraised by two reviewers using the Risk of Bias 2.0 (RoB) tool from Cochrane Collaboration [26]. Where required, authors of papers were contacted to request missing or additional data for clarification. Any disagreements between the reviewers were resolved through discussion. All studies underwent data extraction and synthesis (where possible). Certainty of the evidence was determined using the Grading of Recommendations, Assessment, Development and Evaluation (GRADE) tool [27]. A minimally contextualised assessment of certainty was used, with the null effect being the threshold of interest. [28]

## Results

**Fig. 1:**
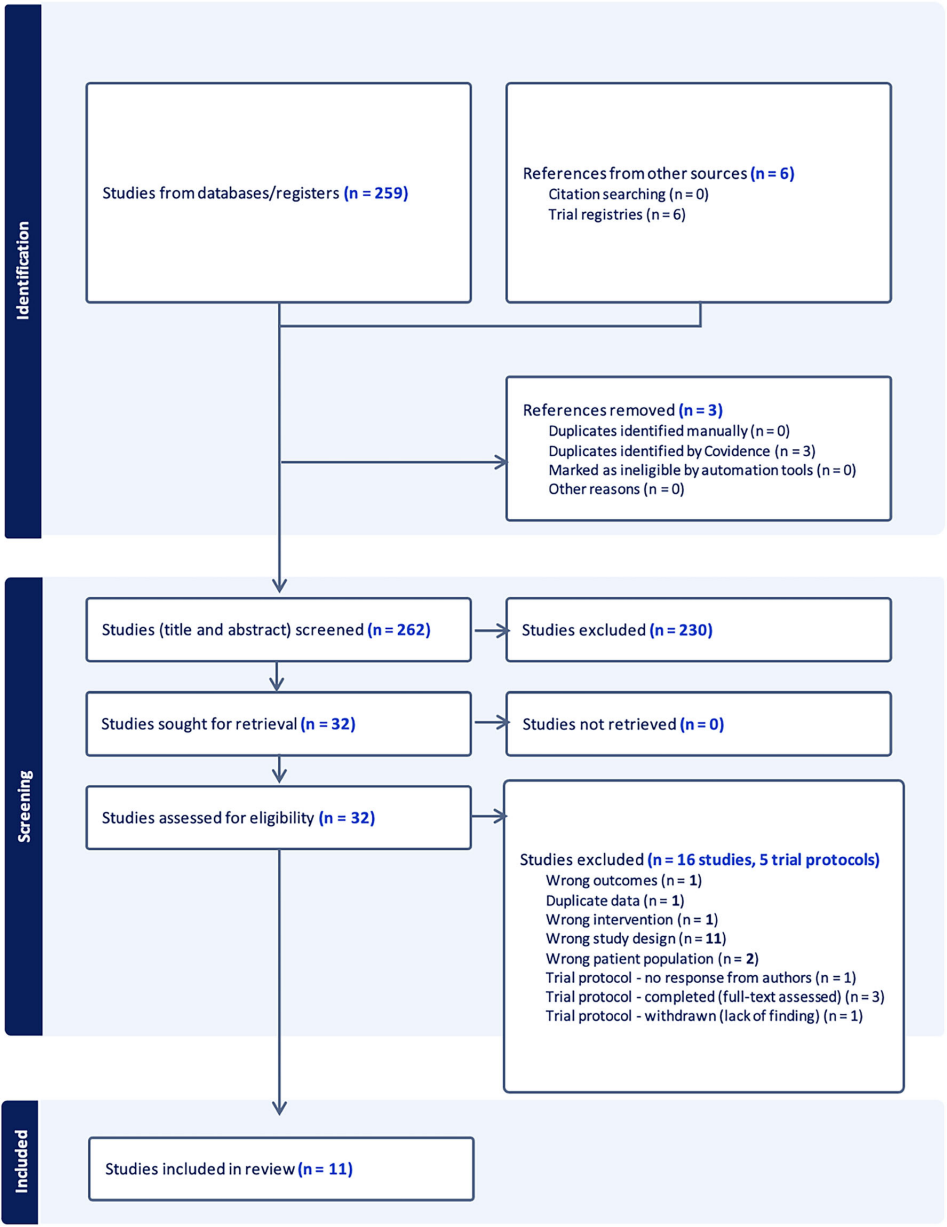
PRISMA flow diagram of included studies

After searching the three databases on 17/04/2022, a total of 259 references were imported into Endnote for screening. An additional six references located from a search of various trial registries, making a total of 265 references. Three duplicate references were removed leaving 262 studies to be screened against title and abstract. After independent screening, 230 studies were excluded, leaving 32 studies meeting full-text screening eligibility. After exclusion of 16 articles and 5 trial protocols during full-text screening, 11 articles [7–11, 18–20, 29–31] were ultimately included for data extraction which may be seen in Fig. 1 below. A refresher database search in June 2024 did not reveal any new articles, apart from the published protocol for this review. [23]

From the included studies, nine were based in Asia and the remaining two from Europe. All studies used a randomised controlled trial study design. Sample sizes were generally small and ranged from 18 to 74 participants. Nine studies [7–11, 18, 20, 29, 31] examined the effects of systemic TXA use and two examined topical TXA use [19, 30]. With regards to surgical experience, where reported, the surgery was done by either a single surgeon [10, 18], an ‘experienced surgical team’ [7, 11, 29], surgeons of ‘similar experience level’ [9], or senior surgical trainees. [8]

Where reported, studies included single jaw [mandibular [8], maxillary [8, 9]] or double-jaw surgery [7, 8, 11, 18, 29, 31]. Most studies reported the use of local anaesthetics with vasoconstrictor agents [9–11, 18, 29], all but two studies reported ‘head-up’ positioning to reduce venous blood loss [18, 20] and all studies aimed to use hypotensive anaesthesia (MAP of <75mmHg or >20% below the preoperative baseline). With respect to outcomes reported, intraoperative bleeding was reported by all, operative time was the second most commonly reported outcome [7–11, 18–20, 30], followed by requirement for blood transfusion [7–10, 18, 29, 30]; changes in haematocrit [7–9, 19] and/or haemoglobin [8, 10, 31] and length of stay [7, 8, 18]. The characteristics of included studies are presented in Table 1.

**Table 1:** Characteristics of included studies

Study (Year)	Study design	Eligibility Criteria	Surgery type	Intervention + Comparison (n)	Outcomes	Adverse Effects
Apipan et al. [7]	RCT	Inclusion: Patients aged 18-35 years with ASA of I Exclusion: Known allergy to TXA, at risk of thromboembolism (OCP), BMI>30, segmental Le Fort I, additional procedure (e.g. genioplasty, angle reduction)	Bimaxillary Osteotomy	G1: 10mg/kg TXA given IV in 100mg 0.9% saline over 30 minutes after induction (20) G2: 15mg/kg TXA at same regimen (20) G3: 20mg/kg TXA at same regimen(20) Co: 100ml 0.9% Saline at same regimen (20)	• Blood loss • Change in Hct • Operative Time • Transfusion • Length of Stay	Nil
Choi et al. [8]	RCT	Inclusion: Patients aged 16-40 years with ASA of I undergoing bimaxillary osteotomy Exclusion: Bone diseases, craniofacial syndromes, other simultaneous non-orthognathic surgeries	Maxilla: Le Fort I with or without segmentalisation or; Anterior maxillary (Wunderer) osteotomy or; Posterior maxillary (Schuchardt) osteotomy or; Le Fort I maxillary distraction. Mandible: Anterior subapical (Hofer) osteotomy Body (Step) osteotomy or; Sagittal split ramus osteotomy or; Vertical subsigmoid osteotomy or; Mandibular body osteotomy and distraction and/or Genioplasty	G1: 20mg/kg TXA given IV in 20ml 0.9% saline over 15 minutes during induction (32) Co: 20ml 0.9% Saline at same regimen (29)	• Blood loss • Change in Hb • Change in Hct • Operative Time • Transfusion • Length of Stay	Nil
Christabel [9]	RCT	Inclusion: Patients aged 18-35 years with ASA of I undergoing Le Fort I Exclusion: Cleft lip and palate, syndromic deformities, pregnancy or lactation, medication that could alter bleeding, coagulopathies, allergy to TXA, intraoperative MAP >70 mmHg	Isolated Le Fort I non-segmented osteotomies	G1: Disjunction + 10mg/kg TXA as IV infusion commenced 30 minutes prior to procedure and continued throughout surgery (50) G2: Tuberosity + 10mg/kg TXA at same regimen (50) Co 1: Disjunction + 0.9% saline at same regime (50) Co 2: Tuberosity + 0.9% saline at same regime (50)	• Blood loss • Change in Hb • Operative Time • Transfusion	Nil
Eftekharian [30]	RCT	Inclusion: Patients with ASA of I, requiring bimaxillary osteotomy Exclusion: Coagulopathy, anticoagulant use or additional non-orthognathic procedures	Bimaxillary Osteotomy	G1: 1g TXA in 1020ml 0.9% saline for tissue irrigation (28) Co: 0.9% Saline for tissue irrigation (28)	• Blood loss • Operative Time • Transfusion	Nil
Jozefowicz [10]	RCT	Inclusion: Patients undergoing a Le Fort I or bimaxillary osteotomies with a haemoglobin >120mg/L and ASA of I-II Exclusion: Haemostasis disorders, anticoagulant or antiplatelet therapy, history of severe renal failure, seizures, or recent thromboembolic events	Le Fort I (10 experimental, 10 control) or Bimaxillary Osteotomy (58 experimental, 58 control)	G1: 1g TXA in 30ml 0.9% saline over 30 minutes during induction, then 1g over 8 hours (74)* Co: 0.9% saline at same regimen (73)**Intention to treat analysis	• Blood loss • Change in Hb • Operative Time • Transfusion	Nil
Kaewpradub [19]	RCT	Inclusion: Patients with ASA of I undergoing bimaxillary osteotomy Exclusion: Anticoagulants	Bimaxillary Osteotomy (75%) +/- genioplasty +/- multi-segmental maxillary osteotomy (25%)	G1: 500mg (in 10ml) TXA in 1000ml 0.9% saline for tissue irrigation (20) Co: 10 mL of 0.9% saline into 1000mL 0.9% saline (20)	• Blood loss • Change in Hct • Transfusion	Nil
Karimi [18]	RCT	Inclusion: Patients aged 18-40 years with ASA of I, undergoing bimaxillary osteotomy Exclusion: Uncontrolled systemic diseases, anticoagulant consumption, simultaneous non-orthognathic surgery (TMJ, craniofacial, autologous bone graft) or bony diseases	Bimaxillary osteotomy (28), with remainder also having genioplasty (4)	G1: 20mg/kg TXA IV before induction of anaesthesia (16) Co: 0.9% saline at same regime (16)	• Blood loss • Operative Time • Transfusion • Length of Stay	Nil
Mortazavi [31]	RCT	Inclusion: Patients with ASA of I undergoing bimaxillary osteotomy Exclusion: Le Fort II, III or genioplasty, bone-related diseases, cleft lips or palates, craniofacial syndromes, concurrent TMJ surgery, bone marrow transplant recipients	Bimaxillary Osteotomy	G1: 20mg/kg TXA IV before induction of anaesthesia (24) Co: 0.9% saline at same regime (24)	• Blood loss (estimate)	Nil
Sankar [29]	RCT	Inclusion: Patients aged 17-30 years with ASA of I Exclusion: Bone diseases, cleft lip and palate, craniofacial syndromes, patients requiring palatal expansion surgery, distraction osteogenesis, simultaneous rhinoplasty, genioplasty, bone graft or implant placement and endoscopically assisted surgeries	Maxilla: • Anterior Maxillary Osteotomy or; • Le Fort 1 without anterior osteotomy Mandible: • BSSO or; • Anterior subapical osteotomy and/or; • Genioplasty	G1: 10mg/kg TXA as IV infusion over 20 mins before incision, then 1mg/kg hourly for duration of surgery (25) Co: 0.9% Saline at same regimen (25)	• Blood loss • Operative Time • Transfusion	Nil
Secher [11]	RCT	Inclusion: Patients aged above 18 years, undergoing bimaxillary osteotomy Exclusion: Pregnancy, history of thromboembolism, cramps, diabetes, connective tissue disorders, cancers, known allergy to TXA, ocular complications, kidney deficiency, or intake of omega-3 fatty acids, garlic, ginseng, and gingko biloba up to 10 days preoperatively	Bimaxillary Osteotomy • Single Maxilla (87) • Segmented Maxilla (9)	G1: 1g of TXA given IV at time of induction (51) Co: 0.9% saline at same regimen (45)	• Blood loss • Operative Time	Nil
Sharma [20]	RCT	Inclusion: Patients aged 15-60 years, with ASA I or II, undergoing Le Fort I and/or BSSO Exclusion: Simultaneous rhinoplasty, coagulopathy, allergy to TXA, abnormal liver/renal function, pregnancy or lactation	Le Fort I Osteotomy (12) or BSSO (11) or Bimaxillary Osteotomy (13)	G1: 15mg/kg TXA infusion over 10 mins during induction + 0.25-0.7 microg/kg/hr of dexmedetomidine during maintenance (18) Co: IV saline infusion over 10 minutes during induction + 0.25-0.7 microg/kg/hr of dexmedetomidine, during maintenance (18)	• Blood loss (estimate) • Operative Time	Nil

RCT: Randomised Controlled Trial; ASA: America Society of Anaesthesiologists Physical Status Classification System; TXA: Tranexamic acid; OCP: Oral Contraceptive Pill; BMI: Body Mass Index; MAP: Mean arterial pressure; BSSO: Bilateral sagittal split osteotomy; TMJ: Temporomandibular joint; (n): Number of participants; G: Group number; Co: Control; IV: Intravenous; Hb: Haemoglobin Hct: Haematocrit

### Risk of Bias of Studies

**Fig. 2:**
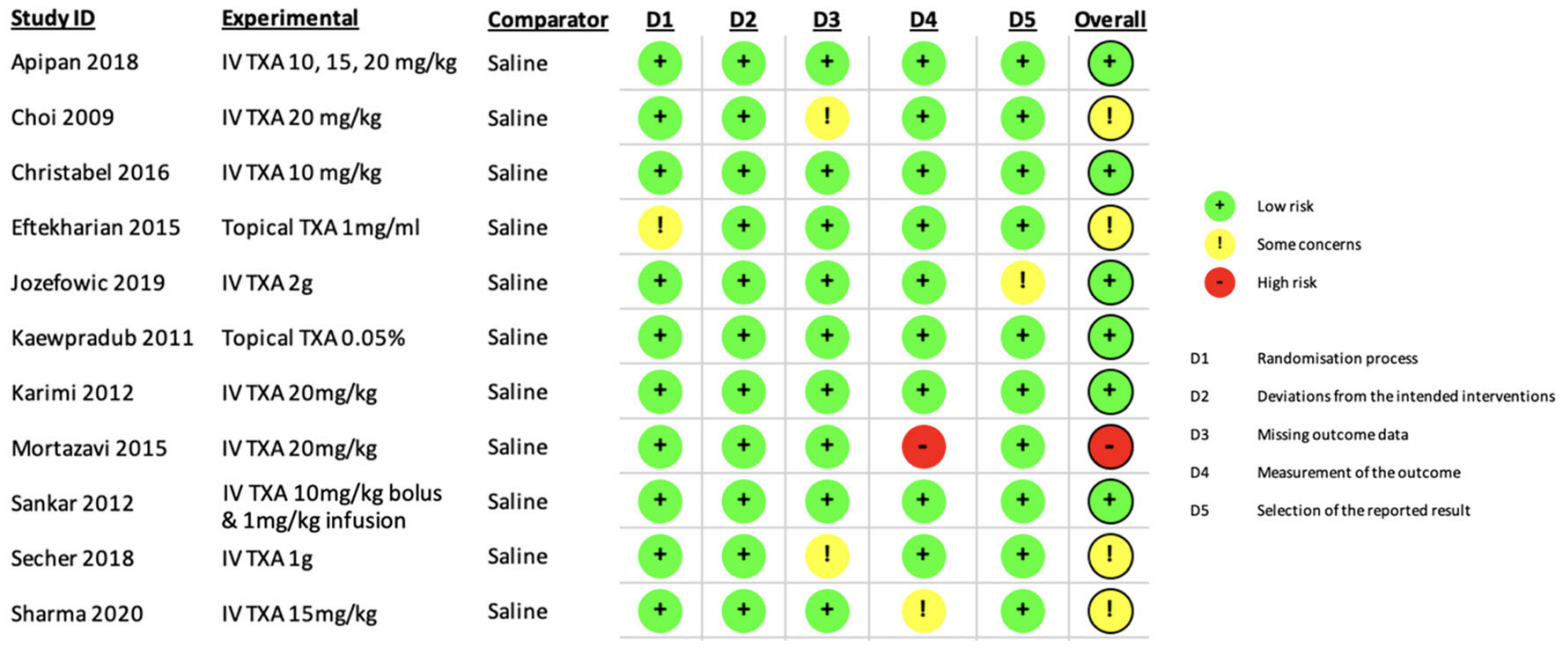
Risk of Bias (RoB) analysis

The risk of bias of studies was mixed when based on RoB 2.0 (Fig. 2). The majority of studies had a low overall risk of bias [7, 9, 10, 18–20, 29], four studies had ‘some concerns’ [8, 11, 30] and one study had a high risk of bias [31]. It should be noted that the study with high risk of bias was published originally in Farsi and was translated into English by medical translators. One study [30] had ‘some concerns’ about the randomisation process, as this was not clearly described. All remaining studies used either computer generated numbers [7, 8, 10, 18, 29] or block method [9, 11] for randomisation.

A single study [10] deviated from the intended intervention, as one of the control patients experienced a significant intraoperative bleed, which required unblinding and subsequent administration of the experimental drug (TXA). This study [10] was consequently graded with ‘some concerns’ in the ‘selection of reported results’ domain, due to the changing allocations of the aforementioned participant. Two studies [8, 11] had missing outcome data as a result of patient dropout, however as investigators were blinded to patient allocation in both studies, they were awarded a grade of ‘some concerns’.

With regards to the Measurement of the Outcome domain, while all other studies had measured and reported intraoperative blood loss volume via volumetric and/or gravimetric means, Mortazavi et al. [31] and Sharma et al. [20] only estimated this outcome using pre-and post-operative haemoglobin. It was agreed by the authors that not reporting the volumetric/gravimetric blood loss was concerning, especially since these data are routinely documented in the operation report. The risk of bias was initially increased to ‘some concerns’ for both these studies and was later elevated to ‘high’ for Mortazavi et al. [31] as it was unclear if the surgeon was blinded to the group allocations. All other outcomes across studies were graded with low risk of bias. The inter-rater agreement (Kappa) was 100%, indicating perfect agreement between reviewers.

### Outcome Analysis

#### Intraoperative Blood Loss

**Fig. 3:**
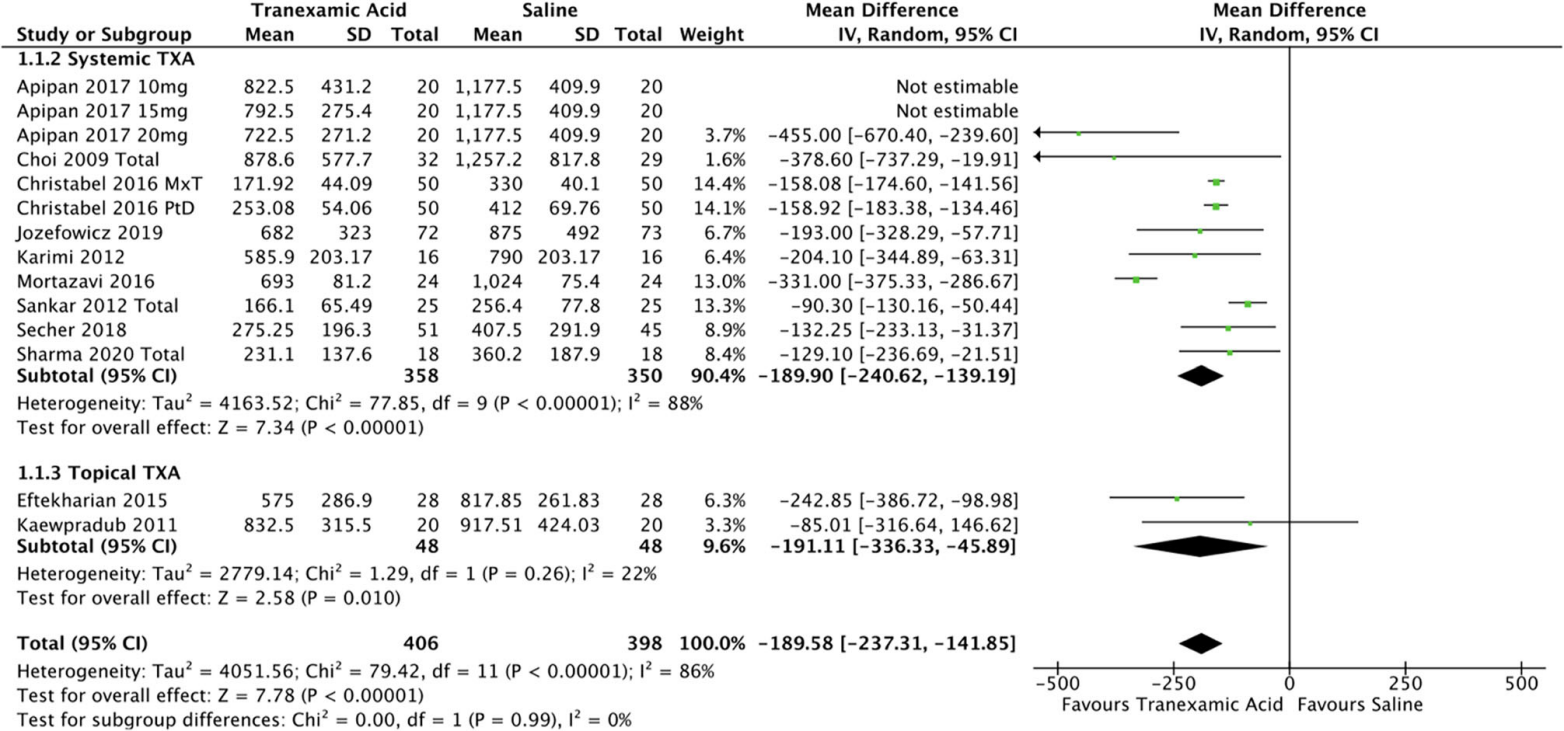
Forest plot for intraoperative blood loss—systemic or topical route. N.B All data from Apipan et al. [7] is presented in the forest plot to show dose-dependent trend; however, it was not pooled in meta-analysis

All 11 included studies reported this outcome. This outcome was measured in two different ways across the studies: volumetric/gravimetric (assuming specific gravity of blood as 1.0, intraoperative blood loss = [volume of fluid in the suction unit—volume of saline using for irrigation] + [weight of blood-soaked gauze—weight of dry gauze] [7–11, 18, 19, 29, 30] or by assessing the change in haemoglobin [20, 31]. Despite this, the same scale (millilitres) was used to describe this outcome in all studies, thus allowing use of weighted mean difference. As evident in the forest plot in Fig. 3 (some data from Apipan et al. [7] is presented in the forest plot to demonstrate a dose-dependent trend, however not pooled into the meta-analysis to avoid double counting), pooled data on 406 participants formed the experimental group and 398 controls, and the use of TXA, at any dosage and in any form, reduced the volume of blood loss by a mean of 189.6 ml (95% CI -237.3, -141.9). While this comparison was statistically significant (P<0.05), as expected, there was significant heterogeneity with *I*^2^ of 86%. The results of nine studies [7–11, 18, 20, 29, 31] (358 experimental and 350 controls) were pooled and showed the use of systemic TXA reduced the intraoperative blood loss by 189.9 ml (95% CI -240.6, -139.2; *p*<0.05; *I*^2^ = 88%). Pooled results of two studies [19, 30] (48 experimental and 48 controls) assessing the effect of topical TXA revealed a reduction in blood loss by 191ml (95% CI -336.3, -45.9; *P*<0.05; *I*^2^ = 22%).

**Fig. 4:**
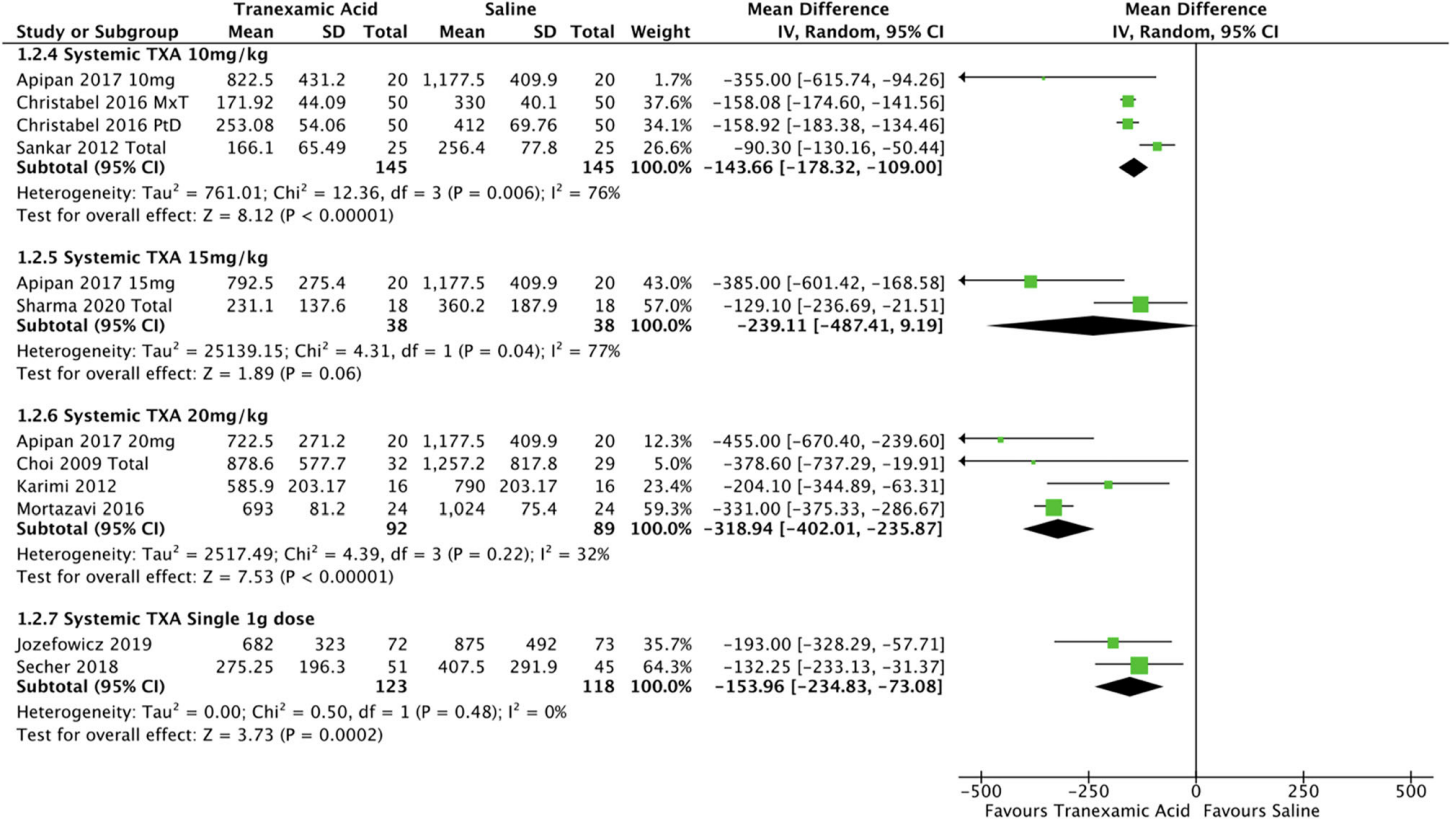
Forest plot for intraoperative blood loss—various systemic dosages

The intraoperative blood loss in various dosages of systemic TXA was also analysed (Fig. 4). In general there were four different dose regimes: 10mg/kg reported in three studies [7, 9, 29] with 145 participants in each arm, 15mg/kg in two studies [7, 20] with 38 participants in each arm, 20mg/kg in four studies [7, 8, 18, 31] with 92 experimental participants and 89 controls, and finally a 1g dose in two studies [10, 11] with 123 in the experimental arm and 118 in the control arm. As evident in the forest plot, the lowest weight-based dose of TXA led to a mean reduction of 143.7ml (95% CI -178.3, -109.0; *P*<0.05; *I*^2^ = 76%). The 15mg/kg dose resulted in a mean reduction of 239.1 ml (95% CI -487.4, 9.2) which was not statistically significant (P>0.05), with high heterogeneity (*I*^2^ of 77%). The 20mg/kg dose reduced mean intraoperative blood loss by 318.9ml (95% CI -402.0, -235.9; *P*<0.05; *I*^2^ = 32%). Finally, the 1.0-gram subgroup analysis yielded a mean reduction of 154.0 ml (95% CI -234.8, -73.1; *P*<0.05; *I*^2^ = 0%).

**Fig. 5:**
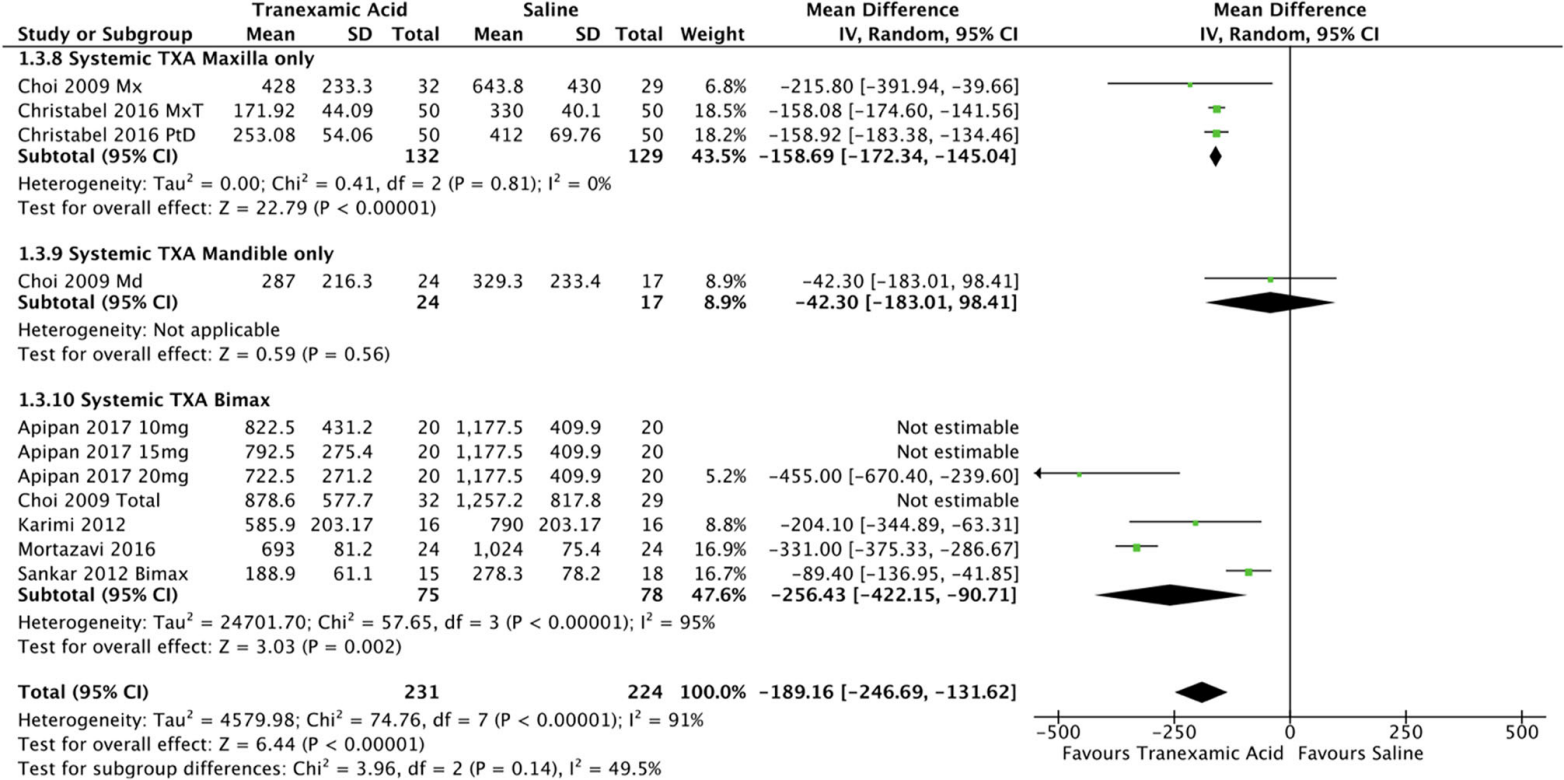
Forest plot for intraoperative blood loss – various surgery types

Finally intraoperative blood loss was analysed based on the type of surgery performed while on systemic TXA (Fig. 5). Two studies [8, 9] with 132 experimental and 129 control participants underwent maxilla-only surgery. Choi et al. [8] additionally reported outcomes for blood loss in mandible-only surgery, and five studies that reported results for bimaxillary surgery [7, 8, 18, 29, 31]. The pooled data for the bimaxillary subgroup included 75 participants in the experimental and 78 in the control arm. The use of systemic TXA in maxilla-only surgery resulted in a mean reduction of blood loss by 158.7ml (95% CI -172.3, -145.0; *P*<0.05; *I*^2^ = 0%). Mean reduction in blood loss was 256.4ml (95% CI -422.2, -90.7; *P*<0.05; *I*^2^ = 95%) was seen in those receiving bimaxillary surgery.

#### Change in Haematocrit

**Fig. 6:**
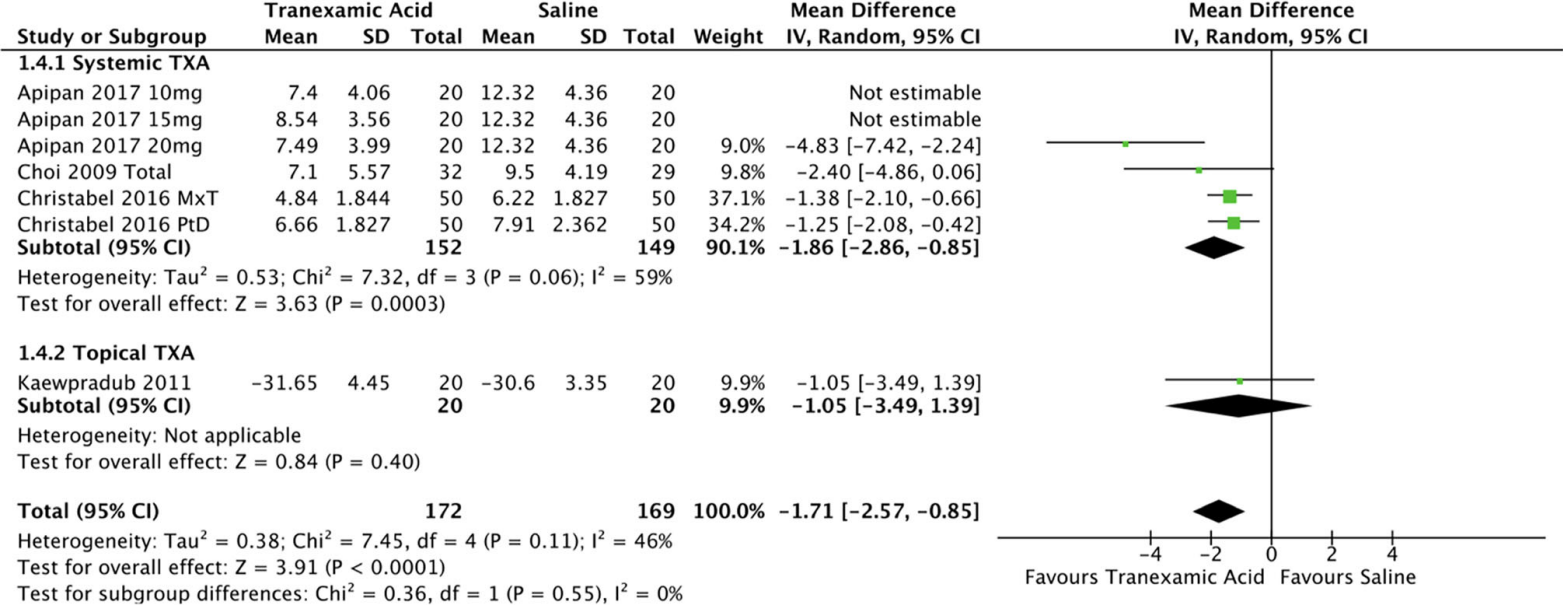
Forest plot for change in haematocrit—systemic or topical route

Data for this outcome were reported in four studies (13-15, 69) [7–9, 19] involving 172 experimental participants and 169 controls. As evident in the forest plot in Fig. 6, the mean difference between the haematocrit drop was 1.7% in favour of the experimental group (95% CI -2.6, -0.85; *P*<0.05; *I*^2^ = 46%).

**Fig. 7:**
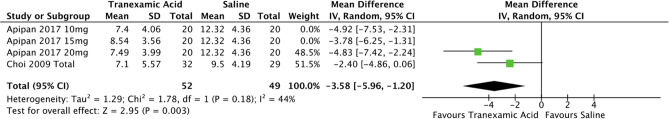
Forest plot for change in haematocrit—various surgery types

Analysis of changes in postoperative haematocrit in studies assessing systemic TXA in bimaxillary surgery was also possible—Fig. 7. Maxilla-only data was only present in one study [9] and there were no studies assessing haematocrit changes in mandible-only surgery. In the bimaxillary surgery analysis, pooled data for 52 experimental participants and 49 controls was available, resulting in a mean difference between the haematocrit drop of 3.6% in favour of the experimental group (95% CI -6.0, -1.2; *P*<0.05; *I*^2^ = 44%).

#### Changes in Haemoglobin

**Fig. 8:**
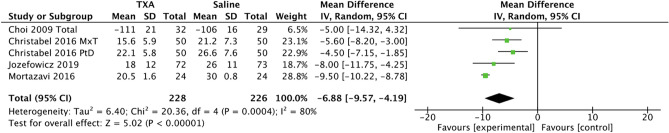
Forest plot for change in haemoglobin—systemic or topical route

Postoperative change in haemoglobin was the third primary outcome. In studies assessing systemic use of TXA, changes in haemoglobin were reported in four studies [8–10, 31], pooling results from 228 experimental and 226 control participants (Fig. 8). The pooled results showed reduction in the haemoglobin drop postoperatively by a weighted mean of 6.9 g/L (95% CI -9.6, -4.2; *P*<0.05; *I*^2^ = 80%).

Choi et al. [8] only presented postoperative haemoglobin mean and standard deviations, and consequently the postoperative haemoglobin figures were converted to negative values (as per the Cochrane handbook [26]) to allow comparison to the other studies that reported changes in haemoglobin.

**Fig. 9:**
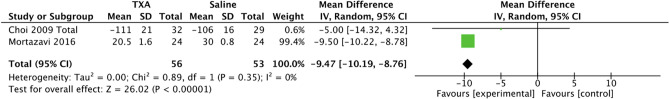
Forest plot for change in haemoglobin—various surgery types (bimaxillary surgery only)

There was only one study reporting haemoglobin change in maxilla-only surgery [9], with no studies in the mandible-only subgroup, and two studies in the bimaxillary surgery subgroup [8, 31]. Analysis of the bimaxillary surgery group was possible for a total of 56 experimental and 53 control participants. The mean difference in postoperative haemoglobin drop was 9.5 g/L (95% CI -10.2, -8.8; *P*<0.05; *I*^2^ = 0%), and is presented in Fig. 9.

#### Rate of Perioperative Blood Transfusion

**Fig. 10:**
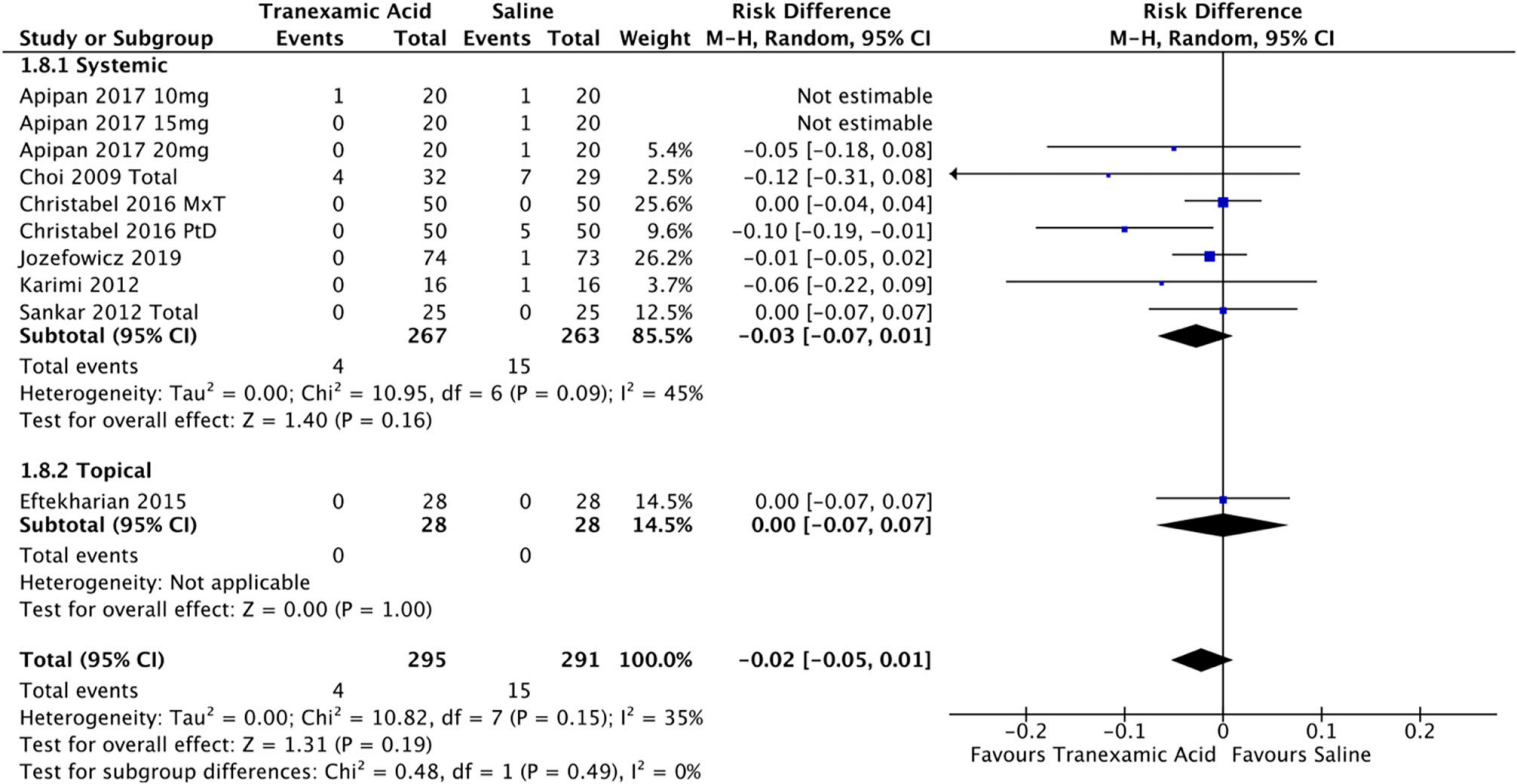
Forest plot for rate of blood transfusion—systemic or topical route

The need for postoperative blood transfusions was the final primary outcome of this review. Data on this outcome was reported in seven studies [7–10, 18, 30], and showed the overall need for transfusion was low: four cases in the experimental group but 15 in the control group (Fig. 10). The risk difference was 2% (95% CI -0.05, 0.01; P>0.05; *I*^2^ of 35%).

#### Operative Time, Length of Stay and Adverse Effects

**Fig. 11:**
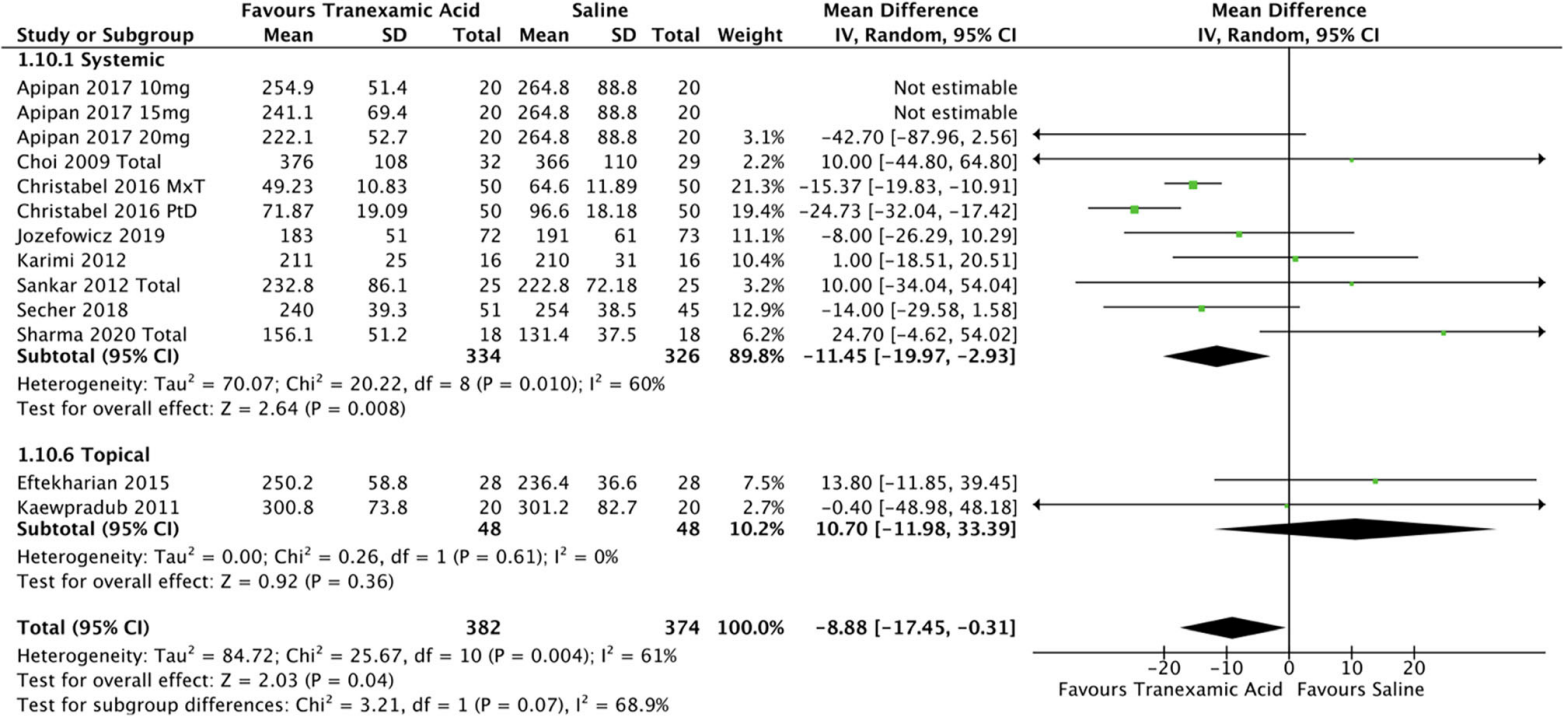
Forest plot for operative time—systemic or topical route

The secondary outcomes of this review included the effect of TXA on operative time, length of hospital stay, and adverse effects. There were no adverse outcomes attributable to TXA use in any of the included studies. The use of TXA in any dosage or route resulted in a mean reduction of operative time by 8.9 minutes (95% CI -17.45, -0.31; *P*<0.05; *I*^2^ = 61%). Data for systemic TXA showed a mean reduction in operation time by 11.45 minutes (95% CI -20.0, -3.0; *P*<0.05; *I*^2^ = 60%) as seen in Fig. 11.

**Fig. 12:**
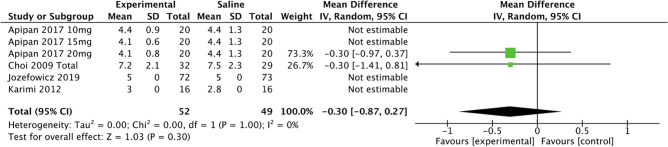
Forest plot for length of stay—systemic route

Four studies [7, 8, 10, 18] showed the mean difference between length of stay was 0.3 days (95% CI -0.9, 0.3; p>0.05; *I*^2^ = 0%) (Fig. 12).

### Sensitivity Analysis

Sensitivity analysis of each outcome, by exclusion of the studies with less-than-ideal risk of bias scores did not significantly change the results.

### GRADE Summary of Findings

A GRADE Summary of Findings table was completed (Table 2). While there were many outcomes of interest, only the seven outcomes of greatest clinical importance are included. Intraoperative blood loss and systemic TXA at any dosage received a high certainty due to low risk of bias, inconsistency, indirectness or imprecision.

**Table 2:**
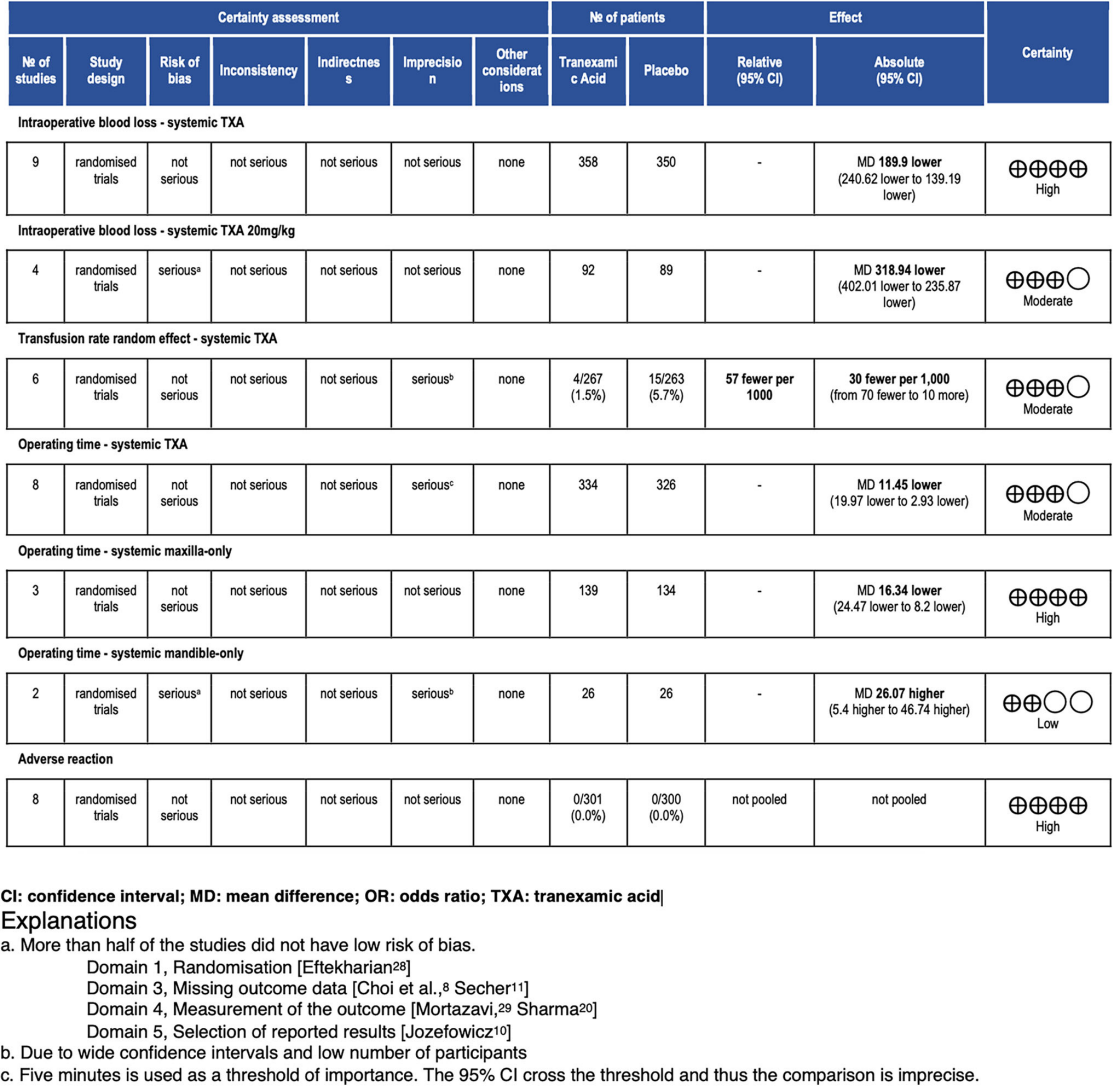
GRADE summary of findings

## Discussion

This systematic review and meta-analysis showed the use of prophylactic TXA in orthognathic surgery resulted in a statistically significant reduction of intraoperative blood loss, irrespective of administration route. When used systemically, dosages of 10 and 20 mg/kg and 1.0 gram resulted in a statistically significant reduction in blood loss volume in maxilla-only and bimaxillary surgery. While a mean reduction of 270ml (seen in the bimaxillary subgroup) may not inherently appear significant, it should be noted that this equates to a 33% reduction in blood loss. [3]

Importantly, Schwaiger et al. [32] suggest that accepted clinical measurements of blood loss—volumetric and gravimetric calculations—provide an underestimate of total blood loss. They reported that calculating the total blood loss based on changes in haematocrit/haemoglobin revealed an additional mean of 287-346ml (additional 32-62%) volume of blood loss from bimaxillary or BSSO surgery. They attributed this hidden volume to haemorrhage into tissue spaces, maxillary sinuses, and ingested blood. While most studies clearly defined the timing of the postoperative data collection (24 hours), one study did not provide details of the specific timing of the postoperative haematocrit [8], and two studies did not provide details of timing of haemoglobin measurement (16, 71) [10, 31]. Most of these studies had systemic TXA rather than topical, so a comparison between the two was unable to be performed.

The rate of postoperative blood transfusion in orthognathic surgery is low, and in turn, the sample sizes were too small to show statistically significant results. Nevertheless, where blood transfusions did take place, there was a trend towards having undergone maxilla-only or bimaxillary surgery. This supports limiting the use of TXA in cases involving maxillary osteotomies.

When considering the impact on operative time, the results of this review suggest that the use of prophylactic TXA, irrespective of route, results in a statistically significant reduction in the operative time. The efficiency of surgery was increased when TXA was used systemically and where maxillary osteotomies were involved. Interestingly, the use of systemic TXA in mandible-only surgery appeared to prolong the operative time; however, this is likely due to the small sample sizes (only 26 participants in each arm) and surgical differences between the two groups.

Nevertheless, reduction of operative time may be attributed to reduced bleeding, the reduced need to achieve haemostasis or create a clear/dry surgical field. Combined, these factors likely contribute to reduced length of hospital stay. Although this was not analysed in this review due inadequate reporting, Andersen et al. showed in their retrospective review that operative time and relative blood loss were predictive of prolonged length of stay. [33]

The findings of this review were in keeping with previous reviews assessing the role of prophylactic TXA in reducing blood loss in orthognathic surgery [34, 35]. When considering the applicability of the interventions in the real world, the regime of one-off systemic TXA dosage at the time of induction, with concurrent use of local anaesthetic, reverse Trendelenburg head position, hypotensive anaesthesia and conventional oscillating saws for osteotomies, is reasonably easy to implement. Tranexamic acid is readily available and associated allergy is rare. The cost of intravenous TXA administration is relatively low [USD$5-50 [36]] compared with costs associated with further haematological monitoring, prolonged surgery or prolonged hospital stay. Finally, systemic TXA use is relatively easy (and convenient) to administer as patients would already have intravenous access for their surgery.

### Strengths and Limitations

This review used a comprehensive search strategy and wide inclusion criterion to capture the relevant literature. By setting no publication date and language limitations across three databases, cross-referencing published trial registries, and screening reference lists of included studies, the risk of missing any relevant peer-reviewed publications was minimised. To enhance the transparency and reproducibility of this review, the protocol was peer-reviewed, published and made publicly available to minimise deviations from the protocol [23]. Nevertheless, there were a few limitations within the search strategy that need to be discussed.

This review utilised clear inclusion and exclusion criteria, and by limiting the inclusion criteria to randomised controlled trials, the highest quality of primary data was utilised. Nevertheless, this strict inclusion criteria likely significantly limited the amount of pooled data. In this review, heterogeneity existed at participant (physiological/anatomical variation), procedural (timing of TXA administration) and surgeon levels (nuances in surgical techniques, varying levels of experience) which cannot be corrected for. Additionally heterogeneity in measurement of outcomes (gravimetric/volumetric vs pre- and post-op haemoglobin/haematocrit) needed to be considered. These variations are evident in the *I*^2^ values for heterogeneity among the studies.

## Conclusion

This systematic review and meta-analysis has demonstrated that the use of prophylactic TXA in orthognathic surgery reduces the intraoperative blood loss, leads to improved post-operative haematocrit and haemoglobin, and reduces the need for blood transfusion. A dosage of 10-20mg/kg or 1g, given intravenously at the time of induction, to healthy patients undergoing orthognathic surgery that involves a LeFort I osteotomy (maxilla-only or bimaxillary osteotomy) reduces operative blood loss and reduces the operative time without any increased risk of thromboembolic events.

## Declarations

### Human or Animal Participants

This article does not contain any studies with human participants or animals performed by any of the authors.
